# Relationship Between Ginsenoside Rg3 and Metabolic Syndrome

**DOI:** 10.3389/fphar.2020.00130

**Published:** 2020-02-25

**Authors:** Hyunji Lee, Gyeyeong Kong, Quangdon Tran, Chaeyeong Kim, Jisoo Park, Jongsun Park

**Affiliations:** ^1^ Department of Pharmacology, College of Medicine, Chungnam National University, Daejeon, South Korea; ^2^ Department of Medical Science, Metabolic Syndrome and Cell Signaling Laboratory, Institute for Cancer Research, College of Medicine, Chungnam National University, Daejeon, South Korea; ^3^ Department of Life Science, Hyehwa Liberal Arts College, Daejeon University, Daejeon, South Korea

**Keywords:** ginsenoside, metabolic syndrome, Rg3, obesity, NAFLD

## Abstract

Metabolic syndrome is an important public health issue and is associated with a more affluent lifestyle. Many studies of metabolic syndrome have been reported, but its pathogenesis remains unclear and there is no effective treatment. The ability of natural compounds to ameliorate metabolic syndrome is currently under investigation. Unlike synthetic chemicals, such natural products have proven utility in various fields. Recently, ginsenoside extracted from ginseng and ginseng root are representative examples. For example, ginseng is used in dietary supplements and cosmetics. In addition, various studies have reported the effects of ginsenoside on metabolic syndromes such as obesity, diabetes, and hypertension. In this review, we describe the potential of ginsenoside Rg3, a component of ginseng, in the treatment of metabolic syndrome.

## Introduction

Ginseng, a traditional herb, is widely used in southeast Asian countries and is gaining popularity worldwide due to its medicinal properties (Takino et al., 1982; Odani et al., 1983). The first record of therapeutic use of ginseng was in Asia about 2,000 years ago (Kim et al., 2015). The roots of ginseng plants are generally used in herbal medicines, but the leaves and fruits are also used (Li W. et al., 2019). Ginseng is produced mainly in Korea, China, and the United States (Karikura et al., 1990; Karikura et al., 1991a; Karikura et al., 1991b), and is distributed as fresh ginseng, dried ginseng, red ginseng, and related products. Ginseng is consumed as a food and health supplement, and is used medically (Lee et al., 2009; Park et al., 2012; Zhao et al., 2016; Lee et al., 2019) due to the presence of ginsenoside saponins, which are pharmacologically active (Matsuda et al., 1986; Peng et al., 2012; Tam et al., 2018; Li G. et al., 2019). As the efficacy of ginseng has been revealed, the corresponding review has been reported (Nakhjavani et al., 2019). About 150 ginsenoside saponins are known, > 90% of which are classified as Rb1, Rb2, Rc, Rd, Re, Rg1, and Rg3. Of these, Rg3, Rg1, Rd, and Rh2 have been studied most intensively (Xie et al., 2015; Xu et al., 2016; Gao et al., 2017; Sun et al., 2017; Zhou et al., 2019). In addition to the segmentation of ginsenoside, saponin components, one ginsenoside may be classified according to its molecular form. Recently, Rg3 has been studied for the different efficacy of two forms. (Kwok et al., 2012; Fan et al., 2017; Nakhjavani et al., 2019)}. Among many ginsenoside saponins, Rg3 is well known in the public, but not much research has been done, such as Rb1, Rg1, and Rb2. In addition, Rg3 is a very low ratio of less than 0.1% among ginsenosides of ginseng. Despite this low ratio, Rg3 has a pharmacological effect such as anticancer, anti-inflammation, and anti-aging that is not much different from that of a high ratio such as Rb1 and Rg1.

## Metabolic Syndrome

Metabolic syndrome is defined as a cluster of metabolic risk factors, such as abdominal obesity, an elevated triglyceride (TG) level, a reduced high-density lipoprotein cholesterol (HDL-C) level, hypertension, and impaired glucose tolerance (van Namen et al., 2019). Approximately 25% of adults worldwide suffer from metabolic syndrome (Nolan et al., 2017; Saklayen, 2018). Metabolic syndrome doubles the risk of atherosclerotic cardiovascular disease and increases that of type 2 diabetes (T2D) fivefold (Grundy, 2016). Abdominal obesity is a major risk factor for metabolic syndrome. As well as being a causal factor in many diseases and disorders, obesity also reduces the quality of life (Finkelstein et al., 2012; Ogden et al., 2014). If the recent trend continues, it is estimated that 57.8% of the world's adult population will be overweight or obese by 2030 (Finkelstein et al., 2012). In addition to obesity, non-alcoholic fatty liver disease (NAFLD) and T2D are also public health issues. NAFLD is currently the most common liver disease in Korea, as well as in the United States and Europe, where its prevalence is 20–30% (Younossi et al., 2018).

Additionally, metabolic syndrome such as obesity, insulin resistance and type 2 diabetes are closely related to chronic inflammation characterized by abnormal cytokine production and activation of the inflammatory signaling pathway network. The first clear link of this association began with the overexpression of tumor necrosis factor-alpha (TNF-α) in the adipose tissue of obese mice (Hotamisligil et al., 1993). In obese mice models, a lack of TNF-α resulted in an improvement in insulin sensitivity and glucose homeostasis, confirming that the inflammatory response plays a critical role in the regulation of insulin action in metabolic syndrome (Uysal et al., 1997; Ventre et al., 1997).

Interest in ginsenoside, a component of ginseng, has increased recently. Among the ginsenosides, Rg3 has been reported to have antiobesity, antidiabetic, antioxidant, anti-aging, anti-inflammatory, and anticancer activity (Takino et al., 1982; Hwang et al., 2009; Kong et al., 2009; Wei et al., 2012; Shin et al., 2013; Sun et al., 2017; Lee et al., 2019). In this review, we focus on the effect of Rg3 on components of metabolic syndrome, including obesity, T2D, hypertension, and NAFLD, as well its therapeutic potential.

## Ginsenoside Rg3

**Figure 1:**
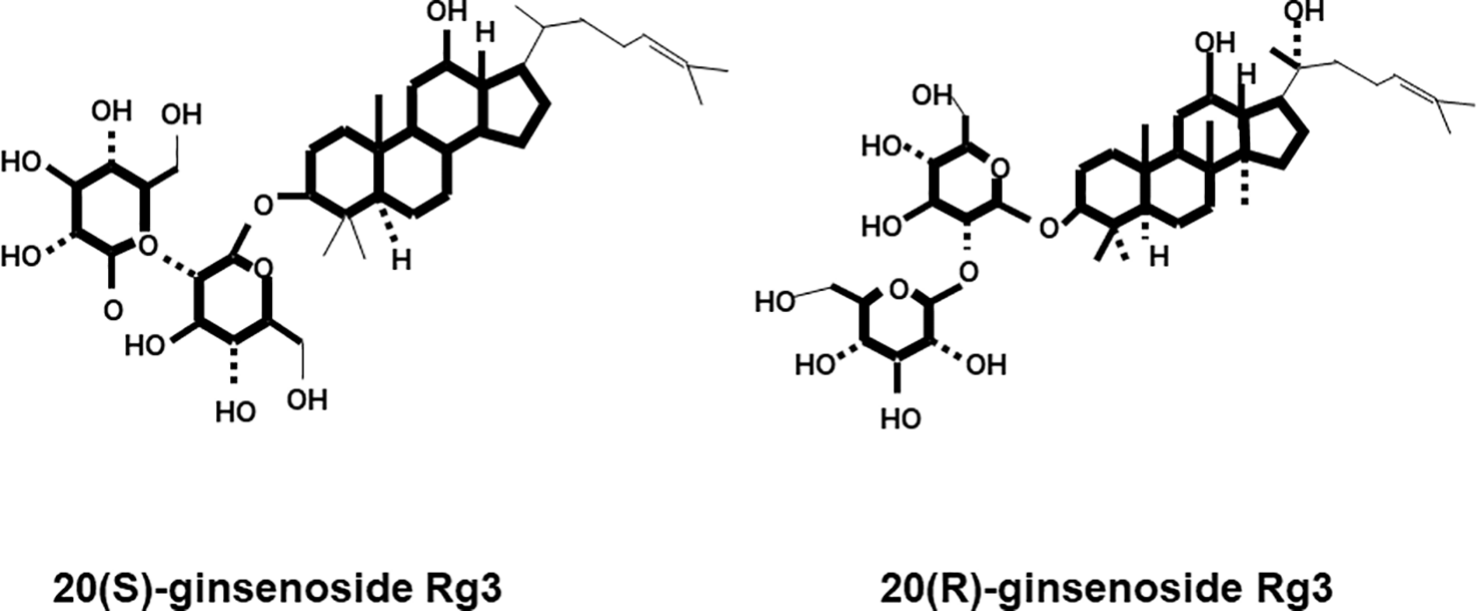
Chemical structures of 20(S)-ginsenoside Rg3 and 20(R)-ginsenoside Rg3.

Ginsenosides are classified according to their aglycon structures: 20(S)-protopanaxadiol (ginsenosides Rb1, Rb2, Rb3, Rc, Rd, and Rg3) and 20(S)-protopanaxatriol (ginsenosides Re, Rg1, Rg2, and Rh1) (Helms, 2004). According to structural differences at the C20 position, there are two enantiomers: the 20(R) and 20(S)-isomers (Kim et al., 2013). For Rg3, there are two structures, 20(R)-Rg3 and 20(S)-Rg3, which are classified according to the C20 position (**Figure 1**). The clinical potential of ginsenosides in various fields of medicine has been investigated.

Ginsenoside Rg3 is one of several biologically active steroid saponin groups found in ginseng and has been shown to exert pharmacological effects. In addition, Rg3, as a natural compound, has antioxidant (Wei et al., 2012), antiaging (Lee et al., 2019), anti-inflammatory (Yoon et al., 2015), and anticancer (Kim et al., 2014; Junmin et al., 2015). Moreover, Rg3 has shown neuroprotective activity in various models (Tian et al., 2005; Shin et al., 2013; Junmin et al., 2015). Rg3 also promotes bone formation and differentiation of preosteoblastic MC3T3-E1 cells (Siddiqi et al., 2015), and inhibits the osteoclastic differentiation of RAW264.7 cells. Rg3 inhibits differentiation by suppressing the RANKL-mediated transcription factors that regulate osteoblastic differentiation (Siddiqi et al., 2015). Also, Rg3 enhances insulin signaling, due primarily to enhanced IRS-1 expression in L6 myotubes (Hwang et al., 2009). Rg3 exerts anti-aging and antioxidant effects in the skin by restoring the function of ultraviolet (UV)-damaged mitochondria, recovering mitochondrial membrane potential and the production of ATP (Lee et al., 2019). Rg3 reportedly inhibits skin photo-aging and light-induced increases in interleukin (IL)-6 and MMP-1 levels, in UV- and infrared (IR)-exposed human dermal fibroblasts and 3D models of normal human skin. Moreover, Rg3 inhibited skin photo-aging and aided the recovery of photo-induced skin damage. (Nagar et al., 2016)

Rg3 is being studied for its effects on various diseases and symptoms. In the following section, we discuss the effects of Rg3 on metabolic syndrome and its therapeutic prospects.

## Effect of Ginsenoside Rg3 in Metabolic Syndrome

Metabolic syndrome refers to metabolic abnormalities associated with visceral adiposity, including hypertension, insulin resistance, dyslipidemia (involving low-density lipoprotein cholesterol [LDL-C], HDL-C, and hypertriglyceridemia), and central obesity (Tariq et al., 2016). Metabolic syndrome is diagnosed when three of five metabolic abnormalities occur simultaneously. Patients diagnosed with metabolic syndrome show damage to tissues in the cardiovascular system, pancreas, and liver (Grundy, 2008; Oladejo, 2011).

## NAFLD

NAFLD is characterized by histological changes similar to alcoholic hepatitis, a disease that is not associated with alcohol abuse in which TGs accumulate in the liver parenchyma (Alba and Lindor, 2003; Angulo, 2003; Wang and Liu, 2003). NAFLD was first discovered in 1980 and ranges from simple steatosis or nonalcoholic fatty liver to nonalcoholic fatty hepatitis (Ludwig et al., 1980). Due to advances in technology, NAFLD can be now diagnosed at an early stage. Nevertheless, NAFLD remains one of the most common liver diseases worldwide (Younossi et al., 1998; Zhou et al., 2012; Lonardo et al., 2013). In addition, as rates of obesity and T2D continue to increase, so too does the incidence of NAFLD. NAFLD is characterized by an increase in intrahepatic triglyceride (IHTG) content (*i.e.,* steatosis), with or without inflammation and fibrosis (*i.e.,* steatohepatitis) (Patel et al., 2016).

The causes of NAFLD are complex and unclear. TGs accumulate in hepatocytes when hepatic lipid influx exceeds lipid export and utilization (Browning and Horton, 2004). This lipid accumulation promotes damage to the liver and leads to hyperinsulinemia and hyperglycemia (Azzout-Marniche et al., 2000; Guillet-Deniau et al., 2002). In addition to indirectly inhibiting free fatty acid (FFA) oxidation when lipid accumulation accelerates the production of new lipid droplets, these FFAs directly damage liver cells and activate inflammatory pathways (Charlton et al., 2002; Klein et al., 2006). Under normal conditions, insulin is secreted in response to circulating glucose and converts FFA into TGs for storage (Feldstein et al., 2003). This normal metabolic process, which is impaired in NAFLD patients, results in high levels of circulating FFAs. In addition, the resulting fatty liver is only vulnerable to hepatotoxicity, which can lead to hepatocellular damage, inflammation, and fibrosis, as well as lipid peroxidation, induction of cytokine production, and mitochondrial dysfunction (Gaemers and Groen, 2006; Duvnjak et al., 2007; Petta et al., 2009; Dowman et al., 2010; Das and Balakrishnan, 2011; Colica and Abenavoli, 2018).

**Figure 2:**
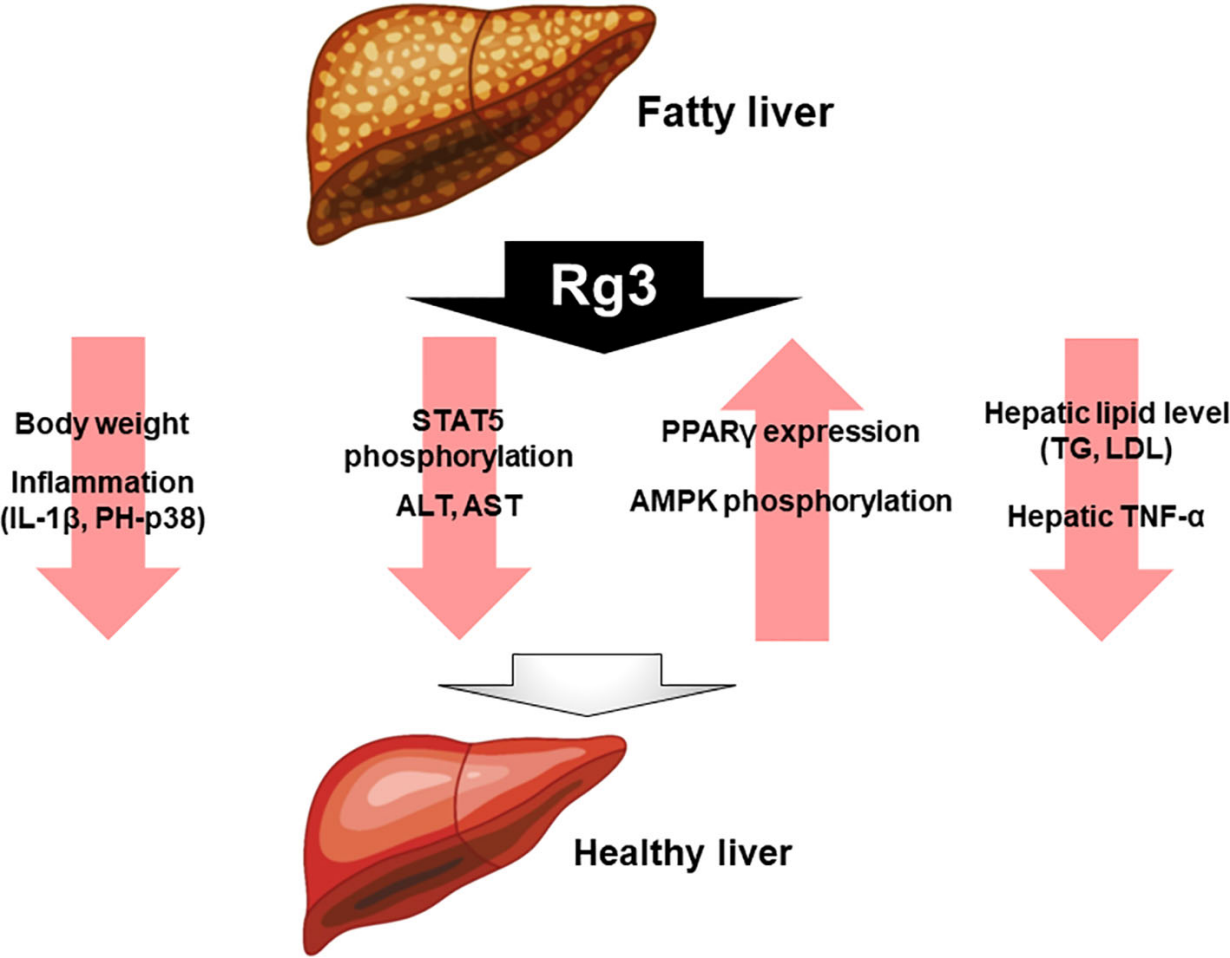
Effect of Rg3 on fatty liver disease. Administration of Rg3 to fatty liver reduces weight loss, inflammation, and reduces liver injury markers alanine aminotransferase and aspartate aminotransferase. In addition, hepatic lipid levels and tumor necrosis factor-alpha expression levels are significantly reduced. On the other hand, PPARγ and AMPK phosphorylation increases, which ultimately improves healthy liver.

Rg3 has been studied as a putative treatment for fatty liver diseases, such as NAFLD. In dyslipidemic and db/db mice, with Rg3 and probiotics improves NAFLD symptoms, reducing liver inflammation by decreasing the expression of cytokines such as IL-1β and phospho-p38 (p-p38) (Kim et al., 2019). In another study, high fat diet-induced mice were compared with a group treated with Rg3 for 8 weeks. The mice treated with Rg3 had lower body weight and better insulin sensitivity compared with the untreated mice. In addition, insulin signaling was higher in the liver and epididymal white adipose tissue. Therefore, Rg3 enhances insulin activity in obesity and T2D models. Furthermore, Rg3 modulates obesity through peroxisome proliferator-activated receptor gamma (PPARγ) regulation, by suppressing signal transducer and activator of transcription 5 (STAT5) phosphorylation (Lee et al., 2017). Rg3 reportedly modulates alanine aminotransferase (ALT) and aspartate aminotransferase (AST) levels, which are used as markers of liver damage. This study analyzed the effects of Rg3 on high fat diet (HFD)-induced ALT and AST levels. Rg3 reduced the incidence of serum postoperative liver failure (PLF), and the hepatic TNF-α level, in high-fat diet-fed mice, and decreased the levels of hepatic lipids including TG and LDL (Nan et al., 2018). In obese insulin-resistant rats, Rg3 increased the PPARγ protein level and adenosine monophosphate-activated protein kinase (AMPK) phosphorylation in the liver (Ginsberg and Maccallum, 2009a). Moreover, Rg3 exerts a positive effect on fatty liver disease (**Figure 2**).

### Obesity

Obesity, which is the major casual factor in metabolic syndrome, is a multifactorial chronic disease caused by genotype-environment interactions (Kissebah and Krakower, 1994). Obesity is a risk factor for insulin resistance and T2D (Lovejoy et al., 1996), as well as a major risk factor for cardiovascular disease (CVD); however, not all obese patients are insulin-resistant or at high risk of diabetes and CVD (Despres et al., 1990). However, excessive visceral fat accumulation increases markers of insulin resistance and the risk of diabetes (Chen and Huang, 2009; Konishi et al., 2010; Ryo et al., 2014; Smith, 2015). Obesity is accompanied by insulin resistance, *i.e.*, impaired glucose tolerance, in which glucose uptake by the muscles is reduced. However, Rg3 increased the expression of the GLUT4 glucose transporter (Kang et al., 2008), and insulin receptor substrate 1 (IRS-1), in obese mice, thereby increasing glucose uptake by the muscles. These mechanisms explain how ginsenoside Rg3-stimulated glucose uptake occurs *via* the PI3K-dependent pathway, of which IRS-1 is a component (Lee et al., 2011). Also, Rg3 exerts glucose- and weight-lowering effects by increasing the GLUT4 protein level in skeletal muscle, and the PPARγ protein level and AMPK phosphorylation in the skeletal muscle of obese insulin-resistant rats (Ginsberg and Maccallum, 2009a).

**Figure 3:**
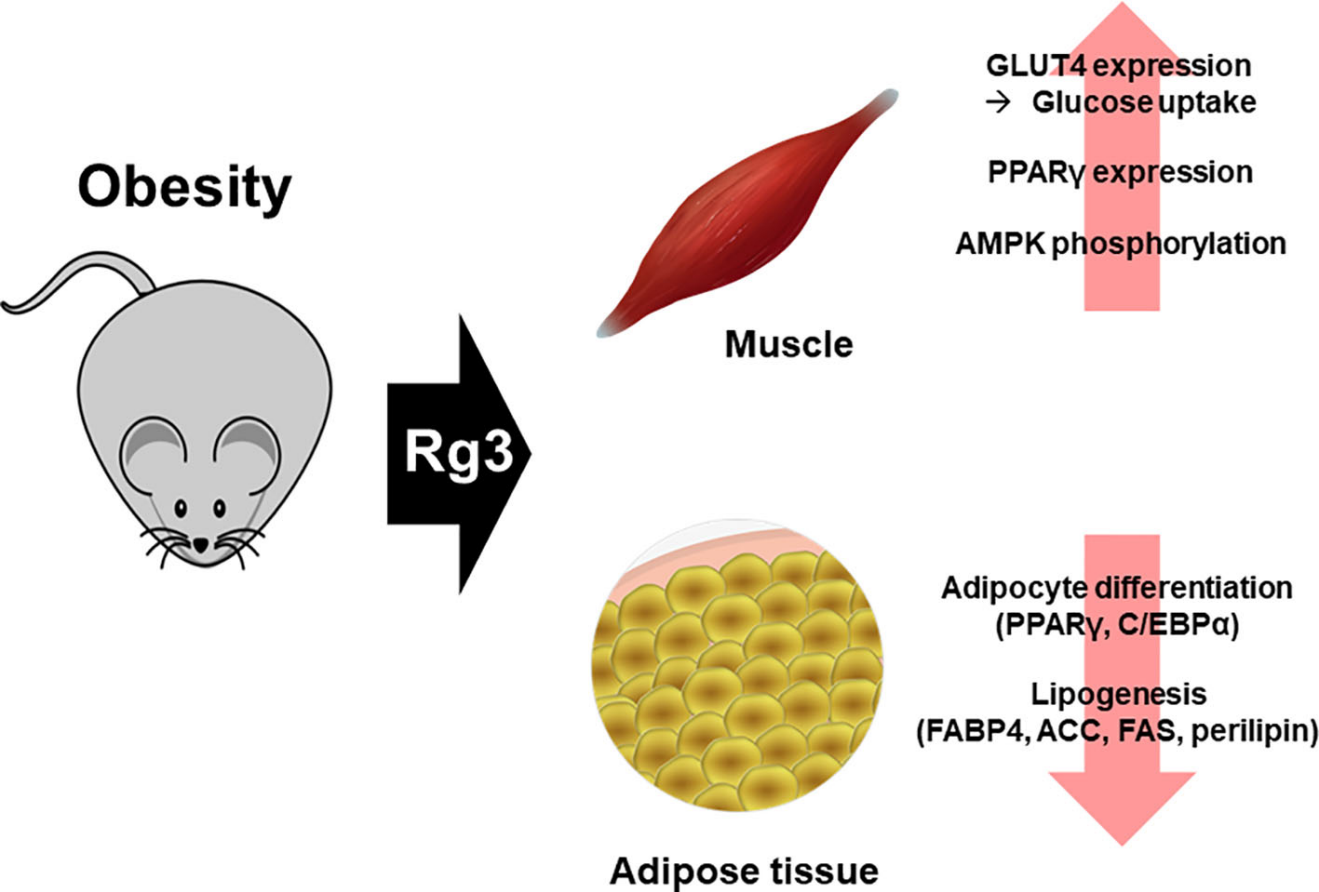
Effect of Rg3 on obesity. Treatment of Rg3 with adipocyte in obese mouse model decreases adipogenesis and lipogenesis. This effect is shown by the reduced expression of PPARγ, C/EBPα, FABP4, ACC, FAS, and perilipin, which are proteins related to lipogenesis. In addition, the glucose uptake is increased by Rg3 and β-oxidation in skeletal muscle of the obese mouse model.

Excessive nutrients are deposited in undesirable locations, such as visceral fat. Nutrients converted to triacylglycerol are used as an energy source, and excess energy is deposited in areas such as the liver, heart, and skeletal muscle (Campaigne, 1990; Bjorntorp, 1991). When these fat tissues are increased due to obesity, the immune system is markedly affected (Ramsay and Caperna, 2009; Maury and Brichard, 2010; Ramanjaneya et al., 2010) and chronic diseases such as heart disease (Leong and Wilding, 1999), diabetes (Cho et al., 2002), and cancer (Basen-Engquist and Chang, 2011) develop. Increased adipose tissue mass due to obesity occurs *via* adipocyte hyperplasia (increased number of adipocytes) and hypertrophy (increased size of adipocytes) (Lee et al., 2013). However, high-fat diet-fed mice treated with Rg3 (20**–**40 µM) not only showed reduced adipose tissue mass, but also inhibition of the production thereof (Hwang et al., 2009; Meng et al., 2018). In addition, treatment of 3T3L1 cells with Rg3 inhibits adipocyte differentiation (Hwang et al., 2009). The fat storage process is closely linked to fat production and breakdown. In particular, the genes involved in fat production, PPARγ and CCAAT enhancer binding protein alpha (C/EBPα), increase the levels of proteins involved in lipogenesis, including FABP4, ACC, FAS, and perilipin. Rg3 also affects adipogenesis and lipogenesis, activates the AMPK pathway (Hwang et al., 2009; Kong et al., 2009), and inhibits the expression of proteins involved in lipogenesis and adipogenesis (Kim et al., 2014). The above effects of Rg3 have been demonstrated *in vitro*, and in obese mouse models (Hwang et al., 2009; Wei et al., 2012; Lee et al., 2017) (**Figure 3**).

### Diabetes

Patients with metabolic syndrome typically die from cardiovascular disease, T2D, or cirrhosis. Ironically, in some parts of the world where malnutrition is common, the incidence of metabolic syndrome has been increasing for decades (Ginsberg and Maccallum, 2009a; Ginsberg and Maccallum, 2009b). T2D is a growing health problem worldwide, and is closely related to the obesity epidemic (Defronzo, 2009; DeFronzo et al., 2015). T2D usually occurs as a result of an imbalance between insulin resistance and secretion (DeFronzo et al., 2015). Individuals with T2DM are at high risk of both macrovascular complications (such as cardiovascular comorbidities) and microvascular complications (including retinopathy, nephropathy, and neuropathy), owing to hyperglycemia and the presence of individual components of insulin resistance (metabolic) syndrome Environmental factors (*e.g.*, obesity, unhealthy diet, and physical inactivity) and genetic factors contribute to several pathophysiological disorders that impair glucose homeostasis in T2D (American Diabetes, 2014; Zitkus, 2014). Increased insulin resistance due to obesity, inflammation, aging, oxidative stress, and decreased physical activity elevate insulin secretion, to overcome insulin resistance and maintain normoglycemia (Kim et al., 2018).

**Figure 4:**
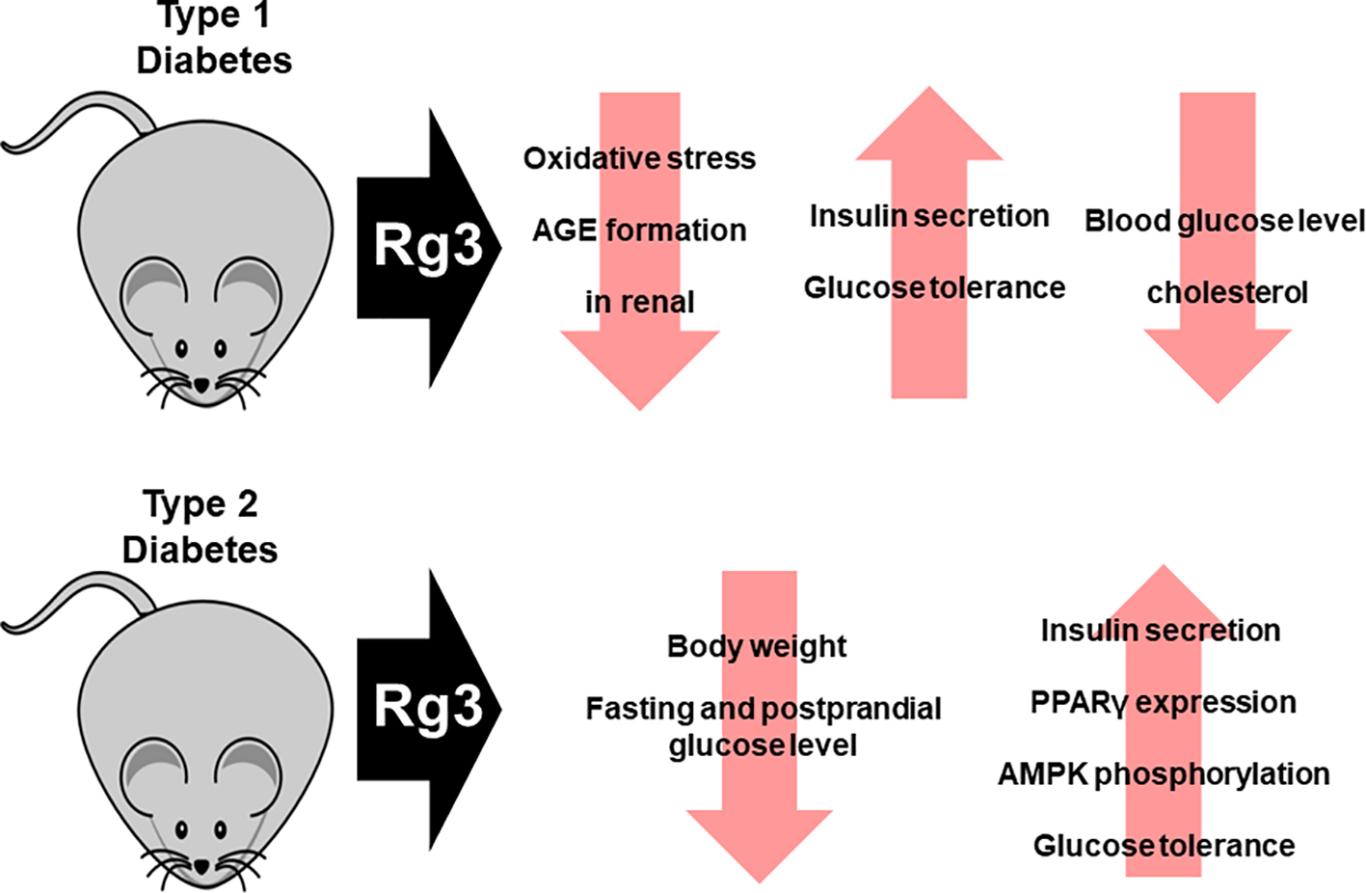
Effect of Rg3 on diabetes. Administration of Rg3 to animal models of type 1 and type 2 diabetes not only recovers impaired renal function but also increases insulin secretion and restores insulin action. In addition, blood glucose levels and cholesterol levels decrease.

In the diabetes-induced Otsuka Long-Evans Tokushima Fatty (OLETF) rat model, Rg3 not only reduced water intake, but also body weight, by inhibiting oxidative stress and advanced glycation end-product (AGE) formation. Therefore, Rg3 improves renal function, which is impaired by T2DM. Similarly, Rg3 exerted a protective effect against type 1 diabetes (streptozotocin-induced diabetic renal damage model) by inhibiting oxidative stress and AGE formation (Yokozawa et al., 2007; Kang et al., 2008). In addition, administration of Rg3 to diabetic OLETF rats has been demonstrated to reduce body weight, as well as fasting and postprandial glucose concentrations (Ginsberg and Maccallum, 2009b). This effect is associated with increased PPARγ expression and AMPK phosphorylation. Rg3 improves insulin secretion, which is important in the treatment of diabetes. Insulin secretion was increased by 20(S)-Rg3 in hamster pancreatic HIT-T15 β-cells in a concentration-dependent manner. Moreover, 20(S)-Rg3 improved glucose tolerance and enhanced insulin secretion in mice with type 1 diabetes (Kang et al., 2008). Moreover, when Rg3-containing malonyl-ginsenosides were administered to streptozotocin-induced diabetic mice at 30 mg/kg, the blood glucose, hepatic glycogen, and cholesterol levels decreased (Liu et al., 2009). Also, Rg3 exerts an anti-hyperglycemic effect in db/db mice by stimulating glucagon-like peptide-1 secretion through the sweet taste receptor-mediated signal transduction pathway (Kim et al., 2015) (**Figure 4**).

### Hypertension

Blood pressure is the force that circulates the blood, putting blood on the walls of the arteries of the body, the main blood vessels of the body (Howard et al., 2003). Hypertension is when blood pressure is too high. It is a serious medical condition that greatly increases the risk of heart, brain, kidney, and other disease. The cause of hypertension are diabetes and obesity due to kidney problems and nerve damage. However, sometimes the cause is unknown (Balcazar-Munoz et al., 2003; Eckel et al., 2006). In particular, hypertension caused by obesity is mostly cause by arteriosclerosis. Obesity causes lipids in the blood to accumulate in blood vessels and harden, resulting in narrowing of bold vessels and development of atherosclerosis (Defronzo, 2006; Ferrannini and Iozzo, 2006).

**Figure 5:**
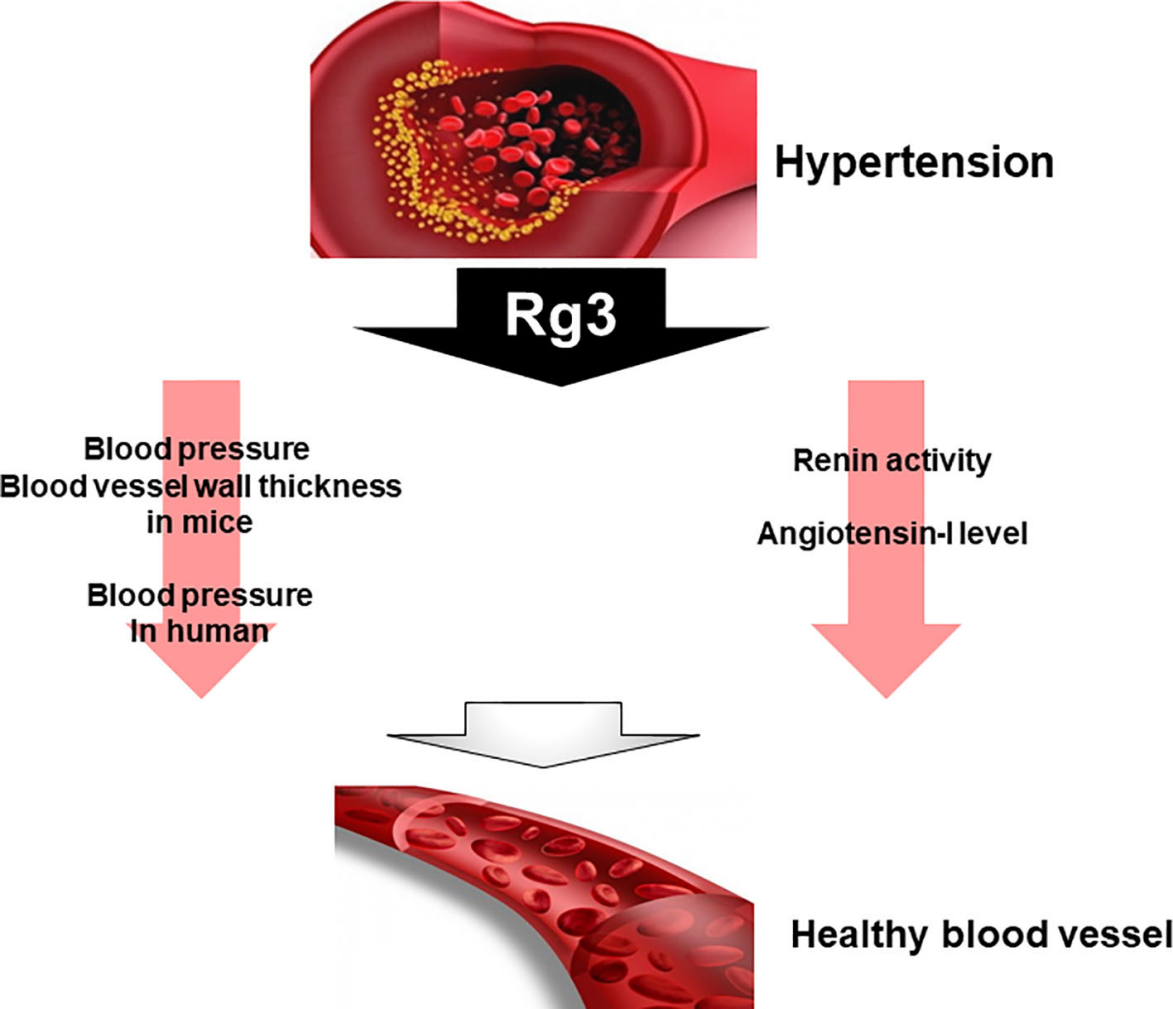
Effect of Rg3 on hypertension. Treatment of Rg3 to hypertension mouse model decreases blood pressure and blood vessel wall thickness in mice and human. In addition, the renin activity and angiotensin-I level also decrease.

In spontaneously hypertensive rats, Rg3 not only significantly reduced renin activity but also decreased blood pressure. However, angiotensin-I converting enzyme inhibition and NO are significantly increased with compared to control (Lee et al., 2016). On the other hand, studies have reported that Rg3 also affects blood pressure in healthy adults. They who consumed Rg3 have decreased central and peripheral arterial pressures (Jovanovski et al., 2014). Another study compared blood pressure and blood vessel wall thickness after Rg3 administration in male mice. Mice treated with Rg3 significantly reduced blood pressure and vascular wall thickness (Nagar et al., 2016). These results suggest that Rg3 can be used as one of the therapeutic agents. Although many studies have been reported on the relationship between metabolic syndrome symptoms and Rg3, there have not been many studies on hypertension and Rg3 among many metabolic syndrome symptoms. Thus, further research is needed on the effects and relationship of Rg3 on hypertension (**Figure 5**).

## Conclusion

Many studies on the association between ginseng and metabolic syndrome have been reported (**Table 1**). However, the relationship between metabolic syndrome and Rg3 requires further study. Interestingly, Rg3 efficacy is subdivided by its molecular forms (S form and R form). But these studies also mainly focus on the pathway of Rg3 action. In addition, in obesity and diabetic animal models, only the effects of RG3 on phenotype are reported. Metabolic syndrome has complex risk factors, but better understanding of Rg3 could lead to a cure. And, based on these studies, no actual clinical treatment is underway. It is urgently necessary to study detailed and logical molecular mechanisms by Rg3. Rg3 studies on islets most closely associated with diabetes and insulin resistance are quite lacing. If research on the effects of Rg3 on islet function and molecular pathway, therapies for type 1 and type 2 diabetes may be proposed. Rg3 may also be incorporated into myotropia caused by diabetes and obesity. If study on satellite cell, not just myoblast and myocyte, is carried out, a prophylactic effect can be expected. For use of Rg3 as a therapeutic agent for obesity and diabetes, the pathways and genes affected by this compound require thorough investigation.

**Table 1:** Summary of effect of Rg3 on metabolic syndrome.

Disease	Observation	Effect	Mechanisms of Rg3 action	Refs.
**NAFLD**	*In vivo* (C57BL/6-HFD, db/db mice)	Reduction of bodyweight and inflammation in liver (IL-1β, Ph-p38)	Inhibition of pro-inflammatory cytokine secretion	(Kim et al., 2019)
	*In vivo* (C57BL/6-HFD)	Reduction of TG level in WAT and liver Decrease hepatic steatosis	Inhibition of PPARγ expression *via* STAT5 phosphorylation suppress	(Lee et al., 2017)
	*In vitro* (3T3L1 cells)	Reduction of lipid accumulation and total TGs		
	*In vivo* (C57BL/6-HFD)	Decrease of serum TC, LDL Decline of TC, TGs, LDL, AST, AST level in liver Increase of serum leptin	Not investigated	(Nan et al., 2018)
	*In vivo* (Otsuka Long-Evans Tokushima Fatty rats)	Increase of PPARγ protein level and AMPK phosphorylation level in liver	Increase of PPARγ protein expression by promoting AMPK phosphorylation	(Ginsberg and Maccallum, 2009a)
**Obesity**	*In vivo* (ICR mice)	Reduce of plasma glucose level	Not investigated	(Kang et al., 2008)
	*In vitro* (HIT-15 cells, C2C12 cells)	Increase of insulin secretion and AMPK phosphorylation		
	*In vitro* (3T3L1 cells)	Increase GULT4 expression level and IRS-1 level Increase glucose uptake	Promotes glucose uptake *via* PI3K-dependent pathway involving IRS-1	(Lee et al., 2011)
	*In vivo* (Otsuka Long-Evans Tokushima Fatty rats)	Decrease of body weight, fasting glucose level and postprandial glucose level Increase of GLUT4 protein level, PPARγ protein level and AMPK phosphorylation level in skeletal muscle	Increase of PPARγ protein expression by promoting AMPK phosphorylation	(Ginsberg and Maccallum, 2009a)
	*In vivo* (C57BL/6-HFD)	Decrease of fat mass and plasma TC, TG level Inhibition of expression lipid synthesis genes Increase of GCK and PGC1-α expression level	Influencing SIRT1 signaling and inhibition of its downstream genes SREBP1c, FAS etc. Accelerate fatty acid β-oxidation and glycolysis pathway	(Meng et al., 2018)
	*In vitro* (3T3L1 cells)	Decrease of PPARγ mRNA level Increase of AMPK phosphorylation level	Not investigated	(Hwang et al., 2009)
	*In vitro* (3T3L1 cells)	Reduction of lipid accumulation Increase of Glycerol secretion Suppress the protein expression of PPARγ, SREBP1c, C/EBPα and perilipin	Not investigated	(Kong et al., 2009)
**Diabetes**	*In vivo* (streptozotocin-induced diabetic renal damage model type 1 diabetes)	Inhibition of oxidative stress and AGE formation Decrease the NF-кB p65, COX-2, iNOS protein levels in renal cortex Reduction of receptors for advanced glycation end product protein levels in renal cortex	Inhibition of NMDA receptor-mediated nitrosative stress	(Kang et al., 2008)
	*In vivo* (streptozotocin-induced diabetic model type 1 diabetes)	Decrease the thiobarbituric acid reactive substance and NF-кB p65, iNOS level in liver Increase of HO-1 protein level in liver Decrease the NF-кB p65, COX-2, iNOS protein levels in renal cortex Decrease the thiobarbituric acid reactive substance and AGE formation	Not investigated	(Yokozawa et al., 2007)
	*In vivo* (Otsuka Long-Evans Tokushima fatty rats)	Decrease of body weight, fasting glucose level and postprandial glucose level	Increase of PPARγ protein expression by promoting AMPK phosphorylation	(Ginsberg and Maccallum, 2009b)
	*In vivo* (streptozotocin-induced diabetic model type 1 diabetes)	Decrease the blood glucose Reduction the hepatic glycogen and cholesterol level	Not investigated	(Liu et al., 2009)
	*In vivo* (db/db mice)	Increase the GLP1 secretion	Stimulation GLP-1 secretion by activating sweet taste receptor signal	(Kim et al., 2015)
**Hypertension**	*In vivo* (spontaneously hypertensive rats)	Reduction of blood pressure and blood vessel wall thickness	Not investigated	(Lee et al., 2016)
	*In vivo* (C57BL/6)	Decrease the renin activity and angiotensin-I level	Not investigated	(Nagar et al., 2016)

## Author Contributions

HL, GK, JiP, QT, and JoP contributed conception and design of the study. HL, JiP, and CK organized the database. HL wrote the first draft of the manuscript. HL, GK, QT, JiP, CK, and JoP wrote sections of the manuscript. All authors contributed to manuscript revision, read and approved the submitted version.

## Funding 

This work was financially supported by a research fund from Chungnam National University (grant to JoP) and by the Brain Korea 21 PLUS Project for Medical Science, Chungnam National University School of Medicine.

## Conflict of Interest

The authors declare that the research was conducted in the absence of any commercial or financial relationships that could be construed as a potential conflict of interest.
